# A systematic review of randomised control trials of sexual health interventions delivered by mobile technologies

**DOI:** 10.1186/s12889-016-3408-z

**Published:** 2016-08-12

**Authors:** Kara Burns, Patrick Keating, Caroline Free

**Affiliations:** 1 Queensland University of Technology, 2 George St, Brisbane, Qld Australia; 2 London School of Hygiene and Tropical Medicine, London, UK; 3 Department Population Health, LSHTM, London School of Hygiene and Tropical Medicine, Keppel St, London, Wc1E 7HT UK

**Keywords:** Sexual Health, Sexually transmitted infection, mHealth, Behaviour change, Randomised controlled trials

## Abstract

**Background:**

Sexually transmitted infections (STIs) pose a serious public health problem globally. The rapid spread of mobile technology creates an opportunity to use innovative methods to reduce the burden of STIs. This systematic review identified recent randomised controlled trials that employed mobile technology to improve sexual health outcomes.

**Methods:**

The following databases were searched for randomised controlled trials of mobile technology based sexual health interventions with any outcome measures and all patient populations: MEDLINE, EMBASE, PsycINFO, Global Health, The Cochrane Library (Cochrane Database of Systematic Reviews, Cochrane Central Register of Controlled Trials, Cochrane Methodology Register, NHS Health Technology Assessment Database, and Web of Science (science and social science citation index) (Jan 1999–July 2014). Interventions designed to increase adherence to HIV medication were not included. Two authors independently extracted data on the following elements: interventions, allocation concealment, allocation sequence, blinding, completeness of follow-up, and measures of effect. Trials were assessed for methodological quality using the Cochrane risk of bias tool. We calculated effect estimates using intention to treat analysis.

**Results:**

A total of ten randomised trials were identified with nine separate study groups. No trials had a low risk of bias. The trials targeted: 1) promotion of uptake of sexual health services, 2) reduction of risky sexual behaviours and 3) reduction of recall bias in reporting sexual activity. Interventions employed up to five behaviour change techniques. Meta-analysis was not possible due to heterogeneity in trial assessment and reporting. Two trials reported statistically significant improvements in the uptake of sexual health services using SMS reminders compared to controls. One trial increased knowledge. One trial reported promising results in increasing condom use but no trial reported statistically significant increases in condom use. Finally, one trial showed that collection of sexual health information using mobile technology was acceptable.

**Conclusions:**

The findings suggest interventions delivered by SMS interventions can increase uptake of sexual health services and STI testing. High quality trials of interventions using standardised objective measures and employing a wider range of behavioural change techniques are needed to assess if interventions delivered by mobile phone can alter safer sex behaviours carried out between couples and reduce STIs.

**Electronic supplementary material:**

The online version of this article (doi:10.1186/s12889-016-3408-z) contains supplementary material, which is available to authorized users.

## Background

### Sexually transmitted infections

Sexually transmitted infections (STIs), including the human immunodeficiency virus (HIV), are a serious worldwide health burden, which if left untreated can lead to a variety of outcomes including cervical cancer, infertility, ectopic pregnancy and mortality [1, 2]. Worldwide it is estimated that half a billion new curable STIs occur each year [3]. Sexual health disease burden varies throughout low, middle and high income countries, with the latest global estimates suggesting an estimated 131 million new cases of chlamydia, 78 million of gonorrhea, 143 million of trichomoniasis and 6 million of syphilis per year [4]. Furthermore, HIV/AIDS ranked six in the top 50 causes of global years of life lost in 2013 [5].

The African region has consistently been reported as having the greatest STI burden with the number of incident cases estimated in 2012 at 37.36 million for trichomoniasis, 12.01 for chlamydia, 11.44 for gonorrhea and 1.84 million for syphilis [4]. In the USA nearly 20 million new STI cases occur every year with direct annual health costs estimated at 16 billion USD [6]. As both developing and developed countries search for new and cost-effective approaches to manage and prevent STIs, there is increasing adoption of electronic and mobile technologies to deliver health promotion, disease prevention interventions and health care services [7, 8].

### mHealth

mHealth can be broadly defined as the use of mobile technologies like mobile phones (standard and smart phones), personal digital assistants, handheld and ultra-portable devices (tablets) and others mobile devices in healthcare to improve healthcare systems, support healthcare professionals and provide better health outcomes for patients [9, 10].

The increased use of mHealth worldwide has grown in parallel to the popularity of the domestic uptake and use of mobile technology, in particular mobile phones. By end of 2015 it has been estimated that there would be more than 7 billion active mobile phone subscriptions with 97 % penetration rate worldwide meaning global mobile phone coverage would surpass all other telecommunications technology [11]. The potential benefits of mHealth using mobiles phones and other devices is being explored globally. In 2011 83 % of the 112 participating World Health Organization Member States reported the presence of at least one mHealth initiative in country, with low-income countries (77 %; *n* = 22) reporting at least one mHealth initiative compared to 87 % (*n* = 29) of high-income countries [12].

### mHealth and STIs systematic reviews

Calls for greater rigor in evaluation has increased the number of mHealth randomised control trials (RCTs) conducted in developed and developing nations. Two previous systematic reviews of controlled trials of m-health interventions from 1999 to 2010 across all health areas (including behaviour change and health service delivery) and regions, identified a total of 117 trials [7, 8]. The reviews found modest benefits for diagnosis and management of health conditions, improvements in smoking cessation and modest increases in attendance with SMS appointment reminders. However, these results should be observed with caution as few of the trials had a low risk of bias. One trial of an adherence to HIV medication intervention showed clinically important reductions in HIV viral load among the intervention group [13]. Three mHealth interventions targeting safer sex behaviours were included in these reviews.

Previous systematic reviews of mHealth interventions for sexual health either targeted a single disease e.g. HIV/AIDS [14, 15] or were specific to Short Message Service (SMS) focusing on only one type of mHealth intervention [16]. The purpose of this systematic review is to update our knowledge of and assess all mHealth interventions for clinic attendance for sexual health and safer sex behaviours (including STI testing, partner notification, condom use number of partners) for all populations, interventions, comparisons, outcomes and studies globally. Interventions designed to increase adherence to HIV medication were not included in the review. This review was conducted by adapting a previously published systematic review protocol [17] for mobile interventions and sexual health and was not registered.

## Methods

### Eligibility criteria

#### Participants

All interventions aimed at patients and the general population were included, with the exclusion of interventions targeting health care professionals and researchers.

#### Interventions

Interventions included all randomised controlled trials utilising mobile technology, including mobile phones, personal digital assistant phones e.g. Blackberry, Palm Pilot, smartphones, enterprise digital assistants, portable media players, e-reader, handheld video-game consoles, handheld and ultra-portable computers such as tablet PCs, smart books and iPads. We excluded desktop personal computers, notebook (laptop) computers, subnotebook computers netbooks, pagers, handheld calculators, pedometers and electronic events-monitoring systems. Additionally, interventions that were mixed mobile technology and non-mobile technology interventions where the treatment and control group both received the mobile technology component, and interventions where there were other treatment differences between the treatment and control groups besides the delivery of the mobile technology components were not included. The focus of this study was on clinic attendance and safer sex behaviours thus studies of adherence to HIV medication were also excluded.

#### Comparisons

Trials were assessed for methodological quality using the Cochrane risk of bias tool and where possible results were converted to intention-to-treat risk ratios and analysed for statistical significance.

#### Outcomes

All outcome measures reported in studies meeting the inclusion criteria were extracted. This included both objective and self-reported measures. Primary outcome measures included any objective measure of health, or health service delivery or use. Secondary outcome measures were defined as self-reported health outcomes relating to knowledge and health-seeking behaviours.

#### Study design

We included all randomised controlled trials. Non-randomised controlled studies were not included.

### Literature search

Including the three trials found in previous studies 1999–2010 [7, 8], we used a three-part search strategy to identify studies meeting the inclusion criteria below that have been published between January 2010 and July 2014: (1) we searched electronic bibliographic databases for published work, using a comprehensive search strategy for mHealth sexual health interventions; (2) we searched trial registers for ongoing and recently completed trials; (3) we searched the reference lists of primary studies included in the review and the reference lists of relevant previously published reviews. This ensured all eligible studies 1999–2014 were included in this review.

The following electronic bibliographic databases were searched: MEDLINE, EMBASE, PsycINFO, Global Health, The Cochrane Library (Cochrane Database of Systematic Reviews, Cochrane Central Register of Controlled Trials, Cochrane Methodology Register, NHS Health Technology Assessment Database, and Web of Science (science and social science citation index). The search strategy only included terms relating to or describing mHealth interventions for sexual health or health service outcomes meeting the inclusion criteria described below.

All of these terms were combined with the Cochrane Library MEDLINE filter for controlled trials of interventions. The mobile technology search terms were adapted for use with other bibliographic databases in combination with database-specific filters for controlled trials, where these are available. There were no language restrictions. Data from dissertations that meet the inclusion criteria, where these are indexed in the above databases, would also be included. We did not retrieve or include any unpublished data. Ongoing, recently completed and unpublished clinical trials meeting the inclusion criteria described were searched for from the following research registers: National Institutes of Health clinical trials registry (USA); National Institute for Health Research Clinical Research Network Portfolio Database (UK); National Research Register Projects Database Archive (UK); and Current Controlled Trials (includes the International Standard Randomised Controlled Trial Number Register).

### Study screening and selection

**Fig. 1:**
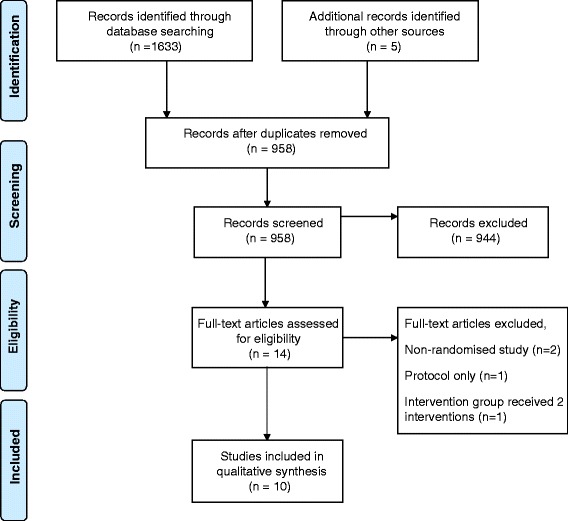
PRISMA 2009 Flow Diagram [40]

Titles and abstracts of studies retrieved using the search strategy and those from additional sources were screened independently by two review authors (KB and PK) to identify studies that potentially met the inclusion criteria. The full text of these potentially eligible studies was retrieved where possible and independently assessed for eligibility by two review authors. Any disagreement between the two review authors over the eligibility of particular studies was resolved through discussion with a third review author (CF). A summary of the data collection process is illustrated in the PRISMA (Preferred Reporting Items for Systematic Reviews and Meta‐Analyses) Flow Diagram (Fig. 1). Additonally the authors prepared the PRISMA 2009 Checklist (Additional file 1).

### Data, quality criteria and data analysis

Two reviewers independently extracted data on the number of randomised participants, intervention, intervention components, behavioural theory informing the intervention, mobile technology employed (e.g. mobile phone/ smartphone), media used (e.g. SMS, Voice message, MMS, application software, telephone), sequence generation, allocation concealment, blinding of outcome assessors, completeness of follow up, evidence of selective outcome reporting, contamination, any other potential sources of bias and on measures of effect using a standardised data extraction form. The authors were not blind to authorship, journal of publication or the trial results. All discrepancies were agreed by discussion with a third reviewer. The behaviour change techniques employed in behaviour change interventions were classified according to Michie’s taxonomy of behaviour change techniques [18]. Risk of bias was assessed according to the Cochrane risk of bias tool. We assessed blinding of outcome assessors and data analysts and we used a cut off of 90 % complete follow up for low risk of bias for completeness of follow up. We contacted study authors for additional information about the included studies, or for clarification of the study methods as required.

All analyses were conducted in STATA v 11. We calculated risk ratios. We planned to use random effects meta-analysis to give pooled estimates where there were two or more trials employing the same mobile technology media (e.g. sms messages) and reporting the same outcome.

## Results

The combined search strategies identified 958 eligible records that were screened for inclusion in the study. Of the 14 potentially eligible studies, 12 full papers and two abstracts were obtained. Of these, ten studies met the inclusion criteria (Fig. 1). The abstract Lim et al. [19] included in Free et al. (2013) [7] review was excluded when the full paper Lim et al. (2012) revealed the intervention group received email and SMS. Similarly, Jones et al. [20] was published in full as Jones et al. [21], with the latter used in this review. Of the ten included trials, there were three intervention categories: 1) promotion of uptake of sexual health services, including reminders to attend a clinic 2) reduction of risky sexual behaviours and 3) reduced recall bias in reporting sexual activity.

### Participants & characteristics of studies

The 10 trials included 16773 participants. Samples ranged from 52 to 7606 participants. Seven trials used a 2-arm design, two a 3-arm and one a 5-arm trial. All trials sought to address STI related issues with two studies focusing on increasing the uptake of testing [22, 23]; two focused on clinic re-attendance [24, 25]; four focused on risk reduction through sexual behaviour change [26–28] one focused on knowledge acquisition *and* risk reduction through sexual behaviour change [29] and one focused on reducing the recall bias when reporting sexual activity [30]. Trials were conducted in high and low-income countries with at-risk populations.

### Interventions

The interventions are described in Table 1–4. For the two studies focusing on increasing the uptake of STI testing one used informational and motivational SMS [22], while the other used a video on a mobile device versus the standard paper-based protocol [23]. SMS reminders were used for the clinic re-attendance trials [24, 25]; one with and without financial incentives [24]. Risk reduction through behaviour change was trialed using SMS [26], video versus SMS [21], informational SMS [27] and informational SMS with theory based feedback and goal setting [28]. One trial focused on knowledge acquisition *and* risk reduction through behavior change used SMS designed for the target population [29]. Finally, one data collection study compared SMS to paper-based and online collection of sexual health information [30]. The maximum number of behavior change techniques employed in interventions was four, the median number of behavior change techniques employed was two. Three interventions reported being developed based on behavioral theory.

### Comparisons

Heterogeneity in interventions and trial outcome assessment and reporting did not allow for meta-analysis.

### Outcomes

The trials reported between one and five outcomes. For primary outcomes, two trials reported outcomes related to clinic attendance [24, 25]. One trial reported uptake of sexual health services [23]. There was also a trial that reported timeliness, completeness and response rate for the use of SMS to collect sexual health information [30]. In regards to secondary outcomes, one trial reported uptake of HIV counselling and testing [22]. Condom use was a common outcome measured among three of the four risk reduction trials [21, 28, 29]. In addition, sexual health knowledge and recent STI testing were also measured [29]. Furthermore, early resumption of sexual activity post circumcision was also reported in one risk reduction trial [27]. Four studies reported measures of acceptability of their interventions [26, 21, 28, 30].

### Study quality

The assessment of study quality is reported in Table 5. No trial had a low risk of bias for all quality criteria.

### Effects

We report the risk ratios for primary outcomes and secondary outcomes. See Tables 6 and 7.

### Uptake of use of sexual health services including increasing testing and clinic re-attendance

#### Primary outcomes

Two trials showed statistically significant increases in clinic attendance in participants receiving clinic reminder SMS compared to controls [24, 25]. Odeny et al. [25] noted a significant decrease in patients that failed to return for a clinic visit (intervention group were more likely to return) after male adult circumcision, relative risk (RR) 0.86, 95 % confidence interval (CI) 0.74–1.00. Downing et al. [24] showed that SMS reminders quadrupled re-testing for Chlamydia compared to controls (RR 4.5, 95 % CI 1.05–19.22). SMS reminder plus incentives had a similar effect as SMS reminders alone. Shahkolahi [23] conducted a 2-arm trial to improve rapid HIV testing in a hospital Emergency Department using videos, a mobile application and paper-based intervention. The authors reported that there was a statistically significant increase in uptake of HIV testing among intervention participants exposed to the mobile application, however, a full paper was not available for this study and risk ratios could not be calculated.

#### Secondary outcomes

One 5-arm trial compared the use of motivational or informational SMS to improve uptake of HIV counselling and testing [22]. Intervention participants either received 3 or 10 motivational/informational SMS. Receipt of informational SMS was not associated with a statistically significant increase in uptake of HIV counseling (RR 0.94, 95 % CI 0.81–1.09 and RR 1.02, 95 % CI 0.89–1.17 for 3 and 10 SMS respectively). However, study participants who received either 3 or 10 motivational SMS were less likely to take up HIV counseling and testing (RR 0.86, 95 % CI 0.73–1.00 and RR 0.8, 95 % CI 0.69–0.93 for 3 and 10 SMS respectively).

### Reduction of risky sexual behaviours including knowledge acquisition and behaviour change

#### Primary outcomes

There were no studies that reported primary outcomes in relation to reduction of risky sexual behaviours.

#### Secondary outcomes

None of the four trials showed statistically significant changes in sexual health behaviours. Gold et al. [29] explored the use of SMS to increase sexual health knowledge and intervention participants scored significantly better in their sexual health knowledge test (RR 1.75, 95 % CI 1.11–2.77) compared to the control group. There were no statistically significant changes in ‘always using condoms in the past 6 months’, (RR 0.87, 95 % CI 0.62–1.24).

Jones et al. [21] compared the effectiveness of HIV prevention messages delivered to smartphones either as weekly messages or through a soap opera video format over a 12-week period. There were no reported statistically significant differences between the two approaches (*p* = 0.39), although reductions in self-reported risky sexual behaviour (*p* <0.001) were reported in each arm compared to baseline at 3 and 6 months’ post intervention. Participants in the trial wanted to continue to receive the videos and reported they could relate to the characters.

Odeny et al. [27] assessed the impact of an SMS intervention to deter early resumption of sexual activity among men who had recently been circumcised. The authors did not find a statistically significant association between receipt of SMS and early resumption of sexual activity (RR 1.13, 95 % CI 0.91–1.38).

Suffoletto et al. [28] investigated the effect of an SMS intervention program to reduce risky sexual behaviour among young women attending an emergency department. No statistically significant differences between intervention and control arms were found for condom use with last vaginal sex (RR 1.4, 95 % CI 0.68–2.88) or for condom use with vaginal sex in the past 28 days (RR 1.4, 95 % 0.49–4.00). In terms of acceptability of the intervention, of the participants who completed the 3-month follow up, all stated that they found the SMS “very informative and very useful.”

Delamere et al. [26] assessed the effect of a 3-month SMS intervention to improve condom usage among young people attending a young person’s clinic. Participants in the intervention group were reported to be almost four times as likely as controls to have changed sexual partner during the study period (RR 3.65, 95 % CI 0.95–14.05), and twice as likely to have unprotected sex, (RR 2.03, 95 % CI 0.47–8.81), but neither result was statistically significant. In terms of acceptability, among intervention participants who were interviewed, 87.5 % reported the text messages useful in their decision making to use condoms, with 19 % of the cohort forwarding SMS to friends. All messages were rated as good, very good or excellent.

### Sexual health data collection to reduce the recall bias when reporting sexual activity

#### Primary outcomes

Lim et al. [30] assessed three methods of sexual health data collection: paper, SMS and online diaries. They found that of the diaries submitted, 80 % of SMS diaries were submitted on the correct day in comparison to 63 % of online diaries.

#### Secondary outcomes

Lim et al. [30] reported 14 measures of acceptability comparing SMS, online and paper diary collection, of which 13 were not statistically significant. The sole statistically significant measure demonstrated that participants were more likely to be uncertain about completing SMS diaries compared to online diaries (*p* = 0.047).

Finally, no subgroup analyses were conducted due to the low number of included studies in this review.

## Discussion

### Key findings

Our systematic review of randomised controlled trials identified 10 RCTs (nine unique study groups) of interventions delivered by mobile technology to improve uptake of services and safer sex behaviours. None of the trials were at low risk of bias. Interventions contained few behavioral change techniques (up to five) and only a third of trials utilised any behaviour change theory in the design of their intervention. Three trials of interventions delivered by mobile phone messaging reported increased uptake of clinic appointments or STI testing. One trial reported increases in knowledge with an intervention delivered by mobile phone messaging. Among the four trials targeting a reduction in risky sexual behaviour, one showed promising increases in condom use, but the trial was small and the findings were not statistically significant. The use of mobile tools to collect sexual health information was shown to be both acceptable, and completed in a timely manner. Small sample sizes of some trials meant they were underpowered to detect effects.

### Strengths and limitations of the review

Our systematic review employed a comprehensive search strategy and we searched 6 data bases and trial registries. Two researchers independently screened abstracts and extracted data from included studies regarding risk of bias and effect estimates. Despite calls for greater rigor in mHealth evaluation in 2008 [31], to date most known reviews have not exclusively focused on randomised studies or applied the Cochrane risk of bias [14, 32]. Our review updates earlier systematic reviews and is the first review focusing on safer sex to describe the content of interventions in terms of the behavioural theories and behaviour change techniques employed [7, 15]. A weakness of this systematic review is that due to the low number of trials reporting similar outcomes and heterogeneity of reporting it was not feasible to calculate pooled effect estimates. Furthermore, it was not possible to perform subgroup analyses due to the low number of included studies. However, as the rate of publication of studies in this area is increasing over time, a future review may be able to overcome this current limitation.

### Discussion of the findings in relation to the existing literature and meaning of findings

This study, in agreement with a previous systematic review, shows that interventions delivered by mobile technology may provide modest benefits in terms of increasing safer sex behaviours carried out by individuals such as increasing clinic attendance and STI testing [7, 8]. It remains unclear if interventions delivered by mobile phone influence safer sex behaviours carried out between couples such as partner notification or condom use. There is a large body of existing research describing a wide range of individual, interpersonal and social and cultural factors influencing (safer) sexual behaviours [33], yet only one intervention delivered by mobile phone aimed to target these influences [21]. Existing face-to-face safer sex interventions which have reported reductions in sexually transmitted infections in randomised controlled trials include up to 19 behaviour change techniques (mean of 12 behaviour change techniques [34–38], whilst the interventions delivered by mobile phone in this review only included up to five behaviour change techniques. The limited number of factors influencing safer sex targeted by interventions, limited use of behavioral theory and limited number of behaviour change techniques employed in interventions are likely to be contributing to the lack of statistically significant findings in trials conducted to date.

## Conclusion

The promising results of improved attendance at clinic appointments due to SMS reminders need to be confirmed in high quality trials. Using standardised objective measures, such as sexually transmitted infection rates, would allow meta-analysis and improve the assessment of any effects of mHealth interventions for sexual health. Studies are needed in low and middle income countries. While mobile phone coverage across the African continent and in many lower and middle-income countries holds promise for delivery of sexual health and other interventions, they remain underrepresented in terms of the quantity of trials conducted.

Additionally, most mHealth interventions are aimed at young populations despite evidence of older populations experiencing an increase in STI transmission [39] and thus future strategies should also consider this group. The results of ongoing trials of safer sex interventions which target a wider range of barriers to safer sex and employ a wider range of behaviour change techniques are needed to determine if interventions delivered by mobile phone can alter behaviours carried out between couples such as partner notification or condom use [34, 35].

## Abbreviations

HBV, hepatitis B virus; HIV/AIDS, human immunodeficiency virus / acquired immune deficiency syndrome; mHealth, mobile health; SMS, short message service; STIs, sexually transmitted infections; UK, United Kingdom; USA, United States of America; USD, United States Dollar

## Appendix

**Table 1:** A description of trials of sexual health interventions delivered by mobile devices

Study	Study Design, mobile technology, and Media	Participants	Aims	Interventions	Comparators
Delamere 2006 [26]	*Study Design:* Parallel group RCT	60 young people aged 17–18 yrs. attending a sexual health clinic.	Determine the acceptability and impact of text messages to promote condom use in adolescents.	Participants received weekly SMS reminding them to use a condom. SMS were written and sent by the study team. Post intervention assessment included a follow up telephone survey. Duration: 3 months.	No Treatment
	*Mobile technology:* Mobile telephone	Control: *n* = 30			
	*Media:* SMS	Intervention: SMS *n* = 30			
	*Country:* Ireland				
De Tolly 2012 [22]	Parallel group RCT; *Mobile technology:* Mobile telephone; *Media:* SMS *Country:* South Africa	2,533 anonymous mobile phone owners. Age data not available.	The aim of this study was to investigate the effectiveness of using SMSs to facilitate uptake of HIV Counselling and Therapy (HCT) in South Africa.	Four intervention groups that received 3 or 10 informational (INFO) or motivational (MOTI) SMSs. After the intervention, participants were prompted to go for HIV Counselling and Testing (HCT). Post-intervention assessment of HIV testing (yes or no) was done after 3 weeks by SMS. Duration: Approx. 2 months	The control group were prompted to go for HIV Counselling and Testing (HCT).
Downing 2013 [24]	Parallel group RCT; *Mobile technology:* Mobile telephone; *Media:* SMS *Country:* Australia	94 patients aged at least 16 years who attended a clinic for treatment of Chlamydia	To assess the effectiveness of using short messaging service (SMS) reminders with and without incentive payments to increase Chlamydia re-testing rates versus the usual care of verbal reminder after initial screening.	Intervention subjects received an SMS reminder for a Chlamydia re-test or an SMS reminder for Chlamydia re-test and a $10 incentive if they returned to the clinic for retesting. Post intervention Chlamydia testing was measured for all participants, (although how it was done was not stated). Duration: Approx. 4 months	The control group received the usual care of verbal reminder after initial screening for a Chlamydia re-test.
		Control: *n* = 32 <25yo = 62.5 %, ≥25 = 37.5 %. Female 56.3 % Aboriginal and Torres Straight Island =31.3 % Non- Aboriginal and Torres Straight Island =62.5 % Not-stated = 6.3 %			
		Intervention: SMS-Only: *n* = 32 <25yo = 56.3 %, ≥25 = 43.7 %. Female 50 %. Aboriginal and Torres Straight Island =28.1 % Non- Aboriginal and Torres Straight Island =59.4 % Not-stated = 12.5 %			
		Intervention: SMS+Incentive: *n* = 30 <25yo = 70 %, ≥25 = 30 %. Female 46.7 %. Aboriginal and Torres Straight Island =26.7 % Non- Aboriginal and Torres Straight Island =73.3 % Not-stated = 0 %			
Gold 2011 [29]	Parallel group RCT; *Mobile technology:* Mobile telephone; *Media:* SMS and MMS *Country:* Australia	7606 people aged 16–29 years who subscribed to a mobile advertising service offered by an Australian mobile telecommunication operator.	To evaluate the use of SMS to (i) evaluate the effectiveness of messages related to safer sex and sun safety and (ii) pilot the use of mobile advertising for health promotion	The intervention subjects were sent a series of eight SMS / MMS sex related healthy behaviour. Duration: 4 months	The control group were sent a series of eight SMS / MMS about sun related healthy behaviour.
		Control: *n* = 3803 (final sample *n* = 200)			
		Range: 16–19yo = 7 % 20–24yo = 35 % 25–29yo = 58 %, Female 40.5 %.			
		Intervention: *n* = 3803 (final sample *n* = 158) Range: 16–19yo = 4 % 20–24 yo = 42.4 % 25–29yo = 53.2 %, Female 39.2 %.			
Jones 2013 [21]	Parallel group RCT; *Mobile technology:* Mobile telephone; phone; *Media:* Video and SMS *Country:* USA	295 women identified as at high-risk of contracting HIV through sex behaviour.	To evaluate the use of SMS versus a 12-episode weekly soap opera video that was created to reduce HIV sex risk behaviour in young urban women.	The intervention subjects were sent weekly trigger emails with videos and received an honorarium of $125 at 3 months and $125 at 6 months. Duration: 6 months	The control group received 12 weekly HIV health promotion written messages over the smartphone and received an honorarium of $125 at 3 months and $125 at 6 months.
		Control: *n* = 146, Mean age 22.0 (SD 3.4)			
		Intervention: *n* = 149, Mean age 22.1 (SD 3.6)			
Lim et al. 2010 [30]	Parallel group RCT; *Mobile technology:* Mobile Smartphone; *Media:* SMS *Country:* Australia	72 participants aged 16–29 who had previously participated in a study about sex/drugs at a music festival.	To evaluate the use of SMS, paper and online diaries of sexual behaviour on response rate, timeliness, completeness of data and acceptability	The participants in the intervention groups completed weekly sexual behaviour diaries for 3 months by SMS and online. Duration: 3 months	The control participants completed weekly sexual behaviour diaries for 3 months on paper that was then submitted by post.
		Control: *n* = 24 Mean age = 20, Female 75 %.			
		Intervention: Online surveys *n* = 24 Mean age = 20, Female 72.7 %.			
		Intervention: SMS *n* = 24, Mean age = 21, Female 69.6 %.			
Odeny 2012 [25]	Parallel group RCT; *Mobile technology:* Mobile telephone; *Media:* SMS *Country:* Kenya	1200 men >18 years who underwent male circumcision.	To evaluated the effect of short message service (SMS) text messages on post-operative clinic visits after adult male circumcision.	Intervention subjects received daily SMS text messages for 7 days on postoperative care and appointment reminders. Duration: 1 mo	Control subjects were advised to return to the clinic within 7 days, but did not receive any SMS messages or a reminder.
		Control: *n* = 600 Mean age =24.8 (IQR 21.5–30.5) Men 100 %			
		SMS: *n* = 600, Mean age =25.0 (IQR 21.4–30.7) Men 100 %			
Odeny 2014 [27]	Parallel group RCT; *Mobile technology:* Mobile telephone; *Media:* SMS *Country:* Kenya	1200 men >18 years who underwent male circumcision.	To examine the effect of text messaging to deter resumption of sex before 42 days post-circumcision	Intervention subjects received usual care (which consisted of HIV testing and counseling, screening and treatment for sexually transmitted infections, condom promotion and provision, risk reduction and safe sex counseling, the MC procedure, and postoperative review) and SMS about postoperative care, appointment reminders and healthy sex behaviours (including abstinence) for the first 7 days and on days 8, 14, 21, 28, 35, 41, and 42 post-procedure. Duration: 2 mo	Control subjects received usual care (which consisted of HIV testing and counseling, screening and treatment for sexually transmitted infections, condom promotion and provision, risk reduction and safe sex counseling, the MC procedure, and postoperative review) only.
		Control: *n* = 600 Range: 18–20yo = 17.8 % 21–30yo = 56.8 % 31–40yo = 16 % >40 = 9.3 %, Mean age = 25.14 (IQR 22.0–31.1), Men 100 %			
		Intervention: *n* = 600, Range: 18–20yo = 17.3 % 21–30yo = 56.4 % 31–40yo = 18.7 % >40 = 7.5 %, Mean age = 25.4 (IQR 22.0–31.2) Men 100 %			
Shahkolahi 2013 [23]	Parallel group RCT; *Mobile technology:* iPad; *Media:* Electronic survey *Country:* USA	450 patients aged 18–70 years from the Howard University Hospital Emergency Department with non-life-threatening illnesses and whose HIV status was either negative or unknown were randomised into two groups. (Median age 35–44 yo), Female 53.1 %	To determine the impact of paper-based and mobile technology-based (iPad) surveys intervention on patients’ desire to receive free rapid HIV screening.	Intervention subjects received the mobile survey and a supplemental video. Duration: 3 mo	Control subjects received the paper-based survey and a supplemental video.
		Control: *n* = 242			
		Intervention: *n* = 208			
Suffoletto 2013 [28]	Parallel group RCT; Mobile technology: Mobile telephone; Media: SMS Country: USA	A convenience sample of 52 female patients (18–25 yo) with hazardous drinking behaviour and recent risky sexual encounters were recruited from an urban Emergency Department.	To examine the effect of a text message (SMS) sex risk reduction program among at-risk young adult female patients discharged from an emergency department (ED).	Intervention subjects were weekly SMS for 12 weeks asking them to report whether they had a risky sexual encounter in the past week. They then received theory-based feedback, and were asked if they were willing to set a goal to refrain from having another risky encounter. Duration: 3 mo	Control subjects received the following SMS for 12 weeks, “Please look for our text in X weeks to complete your web-based follow-up,” where [X] was the number of weeks until study completion.
		Control: *n* = 29 Mean age = 21 (SD 2)			
		Intervention: *n* = 23 Mean age = 22 (SD 2)

**Table 2:** Techniques employed in behaviour change interventions [18, 41]

Behaviour change technique	Number of studies
Goal setting (behaviour)	Suffoletto 2013 [28]; Gold 2011 [29]
Feedback on behaviour	Suffoletto 2013 [28]
Information about health consequences	De Tolly 2012 [22]; Gold 2011 [29]; Odeny 2012 [25]; Odeny 2014 [27]; Suffoletto 2013 [28]
Modelling of the behaviour	Jones 2013 [21]
Social comparison	De Tolly 2012 [22]; Odeny 2014 [27]
Prompts/cues	Delamere 2006 [26]; De Tolly 2012 [22]; Downing 2013 [24]; Gold 2011 [29]; Odeny 2012 [25]; Odeny 2014 [27]; Shahkolahi 2013 [23]
Material incentive (behaviour)	Downing 2013 [24]; Suffoletto 2013 [28]

**Table 3:** Frequency of reported use of behaviour change theory

Behaviour change theory	Number of studies (with their refs from text)
Theory of Planned Behaviour	Gold 2011 [29]
Weinstein’s Precaution Adoption Process model	Gold 2011 [29]
Bandura’s concept of self-efficacy	Gold 2011 [29]
Information-motivation-behavioural skills model of AIDS risk reduction	De Tolly 2012 [22]
Barrett’s power as knowing participation in change theory	Jones 2013 [21]

**Table 4:** List of primary and secondary outcomes of included studies

Study	Primary outcomes	Secondary outcomes
Delamere 2006 [26]	None	Frequency of condom use
		Acceptability of messages
De Tolly 2012 [22]	None	Health seeking behaviour of HIV Counselling & Testing
Downing, 2013 [24]	Health seeking behaviour of chlamydia testing	None
Gold 2011 [29]	None	Changes in sexual health knowledge
		Frequency of condom use
		Health seeking behaviour of STI testing
		Change in number of sexual partners
Jones 2013 [21]	None	Changes through the reduction in unprotected sex with high risk partners
		Acceptability of narratives
		Acceptability of mobile device
Lim et al. 2010 [30]	Response rate,	Acceptability of SMS, online and paper-based diaries
	Timeliness	
	Completeness of data for SMS, online and paper sexual health diaries.	
Odeny 2012 [25]	Health seeking behaviour of clinic attendance	None
Odeny 2014 [27]	None	Avoidance of the resumption of sex before 42 days
Shahkolahi 2013 [23]	Health seeking behaviour of HIV Testing	None
Suffoletto 2013 [28]	None	Sexual Behaviours
		Feasibility
		Acceptability

**Table 5:** Methodological quality summary of interventions and Risk of Bias using the Cochrane Risk of Bias Tool

Trial	Sequence generation	Allocation concealment	Blinding (participants can’t be blinded)	Incomplete outcome data	Selective outcome reporting bias	Contamination	Other bias criteria defined in de Bruin et al. 2015 [42]
Delamere 2006 [26]	Unclear	Unclear	Unclear	High	Unclear	Unclear	Unclear
De Tolly 2012 [22]	Low	Low	High	Low	Low	Low	Unclear
Downing, 2013 [24]	Low	Unclear	High	Low	Low	Low	High
Gold 2011 [29]	Low	Low	High	Unclear	Low	High	High
Jones 2013 [21]	Low	Low	High	Low	Low	Low	Low
Lim et al. 2010 [30]	Low	High	High	Low	Low	Unclear	Low
Odeny 2012 [25]	Low	Low	High	Low	Low	Low	Unclear
Odeny 2014 [27]	Low	Low	High	Low	Low	Low	Unclear
Shahkolahi 2013 [23]	Unclear	Unclear	High	Unclear	Unclear	Unclear	Unclear
Suffoletto 2013 [28]	Low	Unclear	High	Unclear	High	Low	Low

**Table 6:** Measures of effect of primary outcomes

Trial	Intervention	Outcome	RR	95 % CI
Downing, 2013 [24]	SMS reminder for re-testing vs standard advice only	Re-testing for Chlamydia	4.5*	1.05–19.22
Downing, 2013 [24]	SMS reminder for re-testing and financial incentive for re-testing vs standard advice only	Re-testing for Chlamydia	4.27*	0.98–18.51
Odeny 2012 [25]	Educational and reminder SMS messages to promote men who have been circumcised to return for a post-operative visit 7 days	Failure to return for post-operative visit	0.86*	0.74–1.00
Shahkolahi 2013 [23]	Use of video, mobile application and paper based intervention for HIV testing	Rapid HIV testing in the Emergency Department	-^a^	-^a^

^a^ The numbers needed for the calculation were not provided in the paper

* *p* <0.05

**Table 7:** Measures of effect of secondary outcomes

Trial	Intervention	Outcome	RR	95 % CI
Delamere 2006 [26]	Weekly SMS for 3 months	Change of sexual partner	3.66	0.95–14.05
Delamere 2006 [26]	Weekly SMS for 3 months	Unprotected sexual intercourse	2.03	0.47–8.81
De Tolly 2012 [22]	3 informational SMS vs control	Uptake of HIV counseling and testing	0.94	0.81–1.09
De Tolly 2012 [22]	10 informational SMS vs control	Uptake of HIV counseling and testing	1.02	0.89–1.17
De Tolly 2012 [22]	3 motivational SMS vs control	Uptake of HIV counseling and testing	0.86*	0.73–1.00
De Tolly 2012 [22]	10 motivational SMS vs control	Uptake of HIV counseling and testing	0.8*	0.69–0.93
Gold 2011 [29]	SMS on sexual health to increase knowledge	Correct answers in Sexual health knowledge test	1.75*	1.11–2.77
	SMS on sexual health to increase knowledge	Always use condom, past 6 months	0.87	0.62–1.24
	SMS on sexual health to increase knowledge	STI test, past 6 months	1.3	0.83–2.04
Jones 2013 [21]	12-week soap opera videos compared to 12 weekly SMS to reduce dangerous sexual activity	Change in vaginal episode equivalent after 6 months	-^a^	-
Odeny 2014 [27]	Educational and reminder SMS messages to reduce frequency of sexual activity among men 42 days after they have been circumcised	Resumption of sexual activity before 42 days post operation	1.13	0.91–1.38
Suffoletto 2013 [28]	SMS sex risk reduction program	Condom use last vaginal sex	1.4	0.68–2.88
	SMS sex risk reduction program	Condom always used during vaginal sex, past 28 days	1.4	0.49–4.00

^a^ The numbers needed for the calculation were not provided in the paper

* *p* <0.05

## Additional file

Additional file 1: PRISMA 2009 Checklist (DOC 65 kb)
